# App-basierte Anpassung des Cochlea-Implantats durch den Patienten

**DOI:** 10.1007/s00106-024-01544-6

**Published:** 2025-01-09

**Authors:** Andreas Büchner

**Affiliations:** Deutsches Hörzentrum der Medizinischen Hochschule Hannover, Karl-Wiechert-Allee 3, 30625 Hannover, Deutschland

**Keywords:** Prothesen und Implantate, Mobile Anwendungen, Telemedizin, Fernberatung, Telerehabilitation, Prostheses and implants, Mobile applications, Telemedicine, Distance counseling, Telerehabilitation

## Abstract

Heute ist die Versorgung hochgradig Schwerhöriger und ertaubter Patienten mit einem Cochlea-Implantat Routine. Allein an der Medizinischen Hochschule in Hannover werden pro Jahr mehr als 500 Patienten mit einem Cochlea-Implantat (CI) versorgt, d. h. der Patienten-Pool der CI-versorgenden Zentren steigt jedes Jahr signifikant. Weltweit sind mittlerweile etwa 1 Mio. Patienten mit einem derartigen System versorgt, allein in Deutschland schätzt man die Zahl der Versorgten auf über 60.000. Es versteht sich von selbst, dass bei der großen Zahl an CI-Patienten eine zentrale jährliche Routinekontrolle für alle Patienten auf Dauer in den Implantationszentren nicht zu leisten ist. Auch möchten viele Patienten Anreisewege und Zeitaufwand reduzieren, ohne jedoch auf die Kompetenz großer Kliniken verzichten zu müssen. Zur gleichen Zeit steigt der Kostendruck durch die Krankenkassen, und es entsteht die Notwendigkeit, Behandlungen effizienter zu gestalten bei gleicher oder gar besserer Versorgungsqualität. Hier kann und wird die Telemedizin in Form von App-basierter Nachsorge in Zukunft ihren Beitrag leisten.

## Abstract (en)

Treatment of patients with severe hearing loss or deafness using cochlear implants (CI) is nowadays clinical routine. In the Hannover Medical School alone, more than 500 patients are treated with CI annually, meaning that the pool of patients with CI increases significantly each year. Worldwide, there are over 1 million patients with a CI system; in Germany the figure is estimated at over 60,000. With such a huge number of CI patients, centralized annual routine follow-up of all patients in the implant centers is obviously not possible in the long term. Many patients would also like to reduce travel and time expenditures without missing out on the competencies of large hospitals. Simultaneously, the financial pressure exerted by health insurance providers is increasing, resulting in the requirement to provide treatment of the same or even heightened quality more efficiently. Telemedicine in the form of app-based aftercare can help to realize these goals and will play an increasingly important role in the future.

## Heutige Cochlea-Implantatversorgung und ihre Grenzen

Cochlea-Implantate (CI) ersetzen die Funktion der defizienten inneren Haarzellen durch direkte elektrische Stimulation des auditorischen Hörnervs über intracochleäre Mehrkanal-Stimulationselektroden. Aufgrund der schnellen technologischen Entwicklung seit den 1980er-Jahren, insbesondere der Mikroprozessoren, konnten die Hörergebnisse deutlich verbessert werden, sodass die Indikationen für eine Implantation fortlaufend erweitert wurden. Mit weltweit mehr als 1 Mio. implantatversorgter Patienten können CI als Erfolgsgeschichte der Neuroprothetik angesehen werden [1]. Während zu Beginn der 1980er-Jahre nur Patienten mit bilateraler Taubheit als Kandidaten für eine Cochlea-Implantation in Betracht gezogen wurden, werden heutzutage immer mehr Patienten mit Resthörvermögen und Sprachverständnis implantatversorgt.

Die implantierenden Kliniken tragen Verantwortung für den Rehabilitationsprozess der Patienten

Allerdings sind, trotz signifikanter jährlicher Steigerungen der Anzahl an Patienten, bisher nur ein Bruchteil der Patienten, die von einem Cochlea-Implantat profitieren könnten, versorgt. Laut Schätzungen der Weltgesundheitsorganisation (WHO) beträgt die Prävalenz von schwerem Hörverlust (61–80 dB) und Hörminderungen über 80 dB weltweit etwa 1 % bei Erwachsenen [2]. Darauf basierenden Schätzungen zufolge gibt es in Deutschland etwa 1 Mio. potenzielle CI-Kandidaten, von denen derzeit nur etwa 60.000 mit einem Implantat versorgt sind [3]. Die Herausforderung, das wachsende Bedürfnis nach Versorgung mit den vorhandenen Ressourcen in Einklang zu bringen, wird sich mit steigenden Patientenzahlen weiter verschärfen, insbesondere da die implantierenden Kliniken die umfassende Verantwortung für den gesamten Rehabilitationsprozess der Patienten tragen [4].

Dies ist insbesondere so, da die CI-Versorgung nicht nur ein einmaliger chirurgischer Eingriff ist, sondern den Beginn eines lebenslangen Prozesses der akustisch-elektrischen Anpassung und Rehabilitation markiert. Dieser Prozess ist geprägt von einer kontinuierlichen Feinabstimmung des Sprachprozessors, um vorkommende Änderungen in der Hörempfindlichkeit des Patienten in verschiedenen Frequenzbereichen zu kompensieren.

Hier bieten sich die Telemedizin mit Mobile-Health-Applikationen (mHealth) als mögliche Lösung an. Durch die Nutzung von Smartphone-Apps könnten Patienten die Einstellungen ihres CI-Systems eigenständig und in Echtzeit anpassen. Dies würde nicht nur die Unabhängigkeit der Patienten erhöhen, sondern auch die Möglichkeit bieten, feinere Abstimmungen vorzunehmen, die auf spezifische Hörsituationen oder Präferenzen zugeschnitten sind – etwas, das in der Klinik nur sehr bedingt möglich ist.

## Evolution von Fernberatung im Gesundheitswesen

Im weitesten Sinne gibt es Telemedizin seit der Verbreitung der Telefonie, also seit mindestens 120 Jahren. Ärzte gehörten zu den ersten Berufsgruppen, die sich einen Telefonanschluss zulegten, und seit dieser Zeit werden sie Patienten zumindest gelegentlich telefonisch nach ihrem Krankheitsverlauf gefragt haben. Erste medizinische Datenübertragungen gab es bereits 1906 in den Niederlanden. Hier wurden Elektrokardiogramme (EKG) über eine „spezielle Telefonleitung“ zwischen dem Labor des niederländischen Mediziners und Nobelpreisträgers Willem Einthoven und der Leidener Klinik übertragen, da die Apparatur zu groß für einen Einsatz im Krankenhaus selbst war [5]. In den 1970er- und 1980er-Jahren entwickelten sich, vornehmlich in den USA, sog. „Videosprechstunden“, um Menschen in dünn besiedelten Regionen bei Bedarf Kontakt zu Ärzten zu ermöglichen – eine sehr einfache und logische Konsequenz infolge der zunehmenden Verfügbarkeit von Audio- und Videoübertragungsmöglichkeiten jener Zeit.

Telemonitoring ist bereits im Umfeld der Herzschrittmacher und bei der Diabetestherapie üblich

Auch die Fernübertragung von Vitaldaten der Apollo-Astronauten in Echtzeit aus dem All in das Mission Control Center in Houston bereits in den 1960er-Jahren stellt eine Art der Telemedizin dar oder, genauer, des Telemonitorings – etwas, das die Kostenträger gerne in zukünftige Nachsorgemodelle integrieren würden; dieses Vorgehen ist bereits heute im Umfeld der Herzschrittmacher und zunehmend auch bei der Diabetestherapie üblich.

Grundsätzlich teilt sich die Telemedizin in 3 Subbereiche, die wie folgt zu charakterisieren sind, u. a. beschrieben in [6]:

- Echtzeit-Telemedizin,
- Store-and-Forward-Telemedizin (asynchrone Telemedizin),
- Remote Patient Monitoring (RPM).

Echtzeit-Telemedizin als klassische und einfachste Form der Telemedizin stellt die Live-Videokommunikation zwischen Patienten und Gesundheitsdienstleistern dar. Ursprünglich in den 1970er- und 1980er-Jahren in den USA eingeführt, wird sie heute für Routine-Check-up-Untersuchungen, Beratungen und spezialisierte Behandlungen genutzt. Ein gängiges Beispiel sind Videosprechstunden, in denen Ärzte aus der Ferne Diagnosen stellen oder bestehende Therapiepläne anpassen. Diese Form der Telemedizin bietet eine direkte, interaktive Lösung für die Gesundheitsversorgung und ist besonders wertvoll in Gebieten mit begrenztem Zugang zu medizinischen Einrichtungen.

Die Store-and-Forward-Telemedizin (asynchrone Telemedizin) als Form der Telemedizin ermöglicht es, Patientendaten zu einem Zeitpunkt zu erheben und zu einem anderen zu analysieren und auszuwerten. Dabei werden relevante Informationen gesammelt und an gesicherte medizinische Informationssysteme zur weiteren Bearbeitung durch Fachkräfte übermittelt. Besonders vorteilhaft ist diese Methode für den schnellen und sicheren Austausch von Patienteninformationen über verschiedene geografische Standorte hinweg. Store-and-Forward wird häufig in Fachbereichen wie Radiologie, Augenheilkunde, Dermatologie und Pathologie eingesetzt, wo bildbasierte Daten eine zentrale Rolle spielen. Grundsätzlich kann das Verfahren aber auch bei anderen Datentypen eingesetzt werden.

Remote Patient Monitoring (RPM) stellt eine Schlüsselkomponente in der modernen Gesundheitsversorgung dar, insbesondere bei der Betreuung von Patienten mit chronischen Krankheiten wie Diabetes oder Herzleiden. Durch RPM können medizinische Fachkräfte Patientendaten aus der Ferne überwachen, im Bedarfsfall sogar in Echtzeit. Dies ermöglicht eine kontinuierliche Überwachung des Gesundheitszustands und eine schnellere Reaktion auf eventuelle Veränderungen. Ein Beispiel hierfür ist die Überwachung des Blutzuckerspiegels bei Diabetikern, bei der Messwerte automatisch an ein Expertensystem übertragen werden, um Tendenzen rechtzeitig zu erkennen und eine optimale Behandlung zu gewährleisten.

mHealth sind mobile Anwendungen, die die Gesundheitsüberwachung in die Hände der Patienten legen

Die Evolution der Telemedizin in den letzten Jahrzehnten spiegelt die rasante Entwicklung der Informations- und Kommunikationstechnologie wider. Mit dem Aufkommen des Internets und mobiler Technologien hat sich die Telemedizin weiterentwickelt und eHealth sowie mHealth hervorgebracht. Während der Begriff eHealth das breite Spektrum digitaler Gesundheitsdienste umfasst, einschließlich elektronischer Gesundheitsakten und Online-Portalen, konzentriert sich mHealth speziell auf mobile Anwendungen, die die Gesundheitsüberwachung und -kommunikation direkt in die Hände der Patienten legen.

Einhergehend mit dem technischen Fortschritt besitzt die Mehrzahl der Bevölkerung heute ein Smartphone, welches die Rechenleistung von Supercomputern des letzten Jahrhunderts bei Weitem übertrifft. Deep Blue, der 1997 als erster Computer einen amtierenden Schachweltmeister schlug, hatte etwa 11 GIGAFLOPS („floating point operations per second“, Gleitkommaoperationen pro Sekunde) Rechenleistung. Ein heutiges Smartphone im Wert von 300 € durchbricht die Ein-TERAFLOPS-Marke deutlich und kann damit etwa 100-mal schneller rechnen als der schrankgroße Deep Blue. Mit mHealth-Applikationen auf Smartphones kommt seit einigen Jahren Bewegung in die Telemedizin: Blutdruck, Blutzucker, Temperatur, Fitnessdaten, EKG – immer mehr Menschen „vermessen“ sich selbst und speichern ihre Daten „in der Cloud“. Sie tun dies teilweise aus Interesse an dem technisch Machbaren, oftmals aber auch aus der medizinischen Notwendigkeit heraus, Vitaldaten für den nächsten Arztbesuch mit einem zertifizierten Medizinprodukt zu erheben – etwa um Herzrhythmusstörungen zu dokumentieren.

Während Telemedizin, eHealth und mHealth dabei sind, die Gesundheitsversorgung zu revolutionieren, stehen sie gleichzeitig vor neuen Herausforderungen und Chancen. Datenschutz und Sicherheit, insbesondere bei der Speicherung und Übertragung sensibler Gesundheitsdaten, sind zentrale Themen. Andererseits können diese Technologien helfen, die personalisierte Medizin und die präventive Gesundheitsversorgung deutlich zu forcieren. Die fortschreitende Integration von künstlicher Intelligenz (KI) und maschinellen Lernens in mHealth-Anwendungen wird auf der Basis wachsender Datenbestände zu noch präziseren Diagnosen und individualisierten Behandlungsplänen führen.

Dieser Trend, der in anderen Bereichen der Medizin bereits Fuß gefasst hat, nimmt nun auch im Bereich der zukünftigen CI-Nachsorge Formen an.

## App-basierte Nachsorgekonzepte für CI-Patienten

Die konsequente Nutzung von Selbstnachsorge-Apps könnte die Rehabilitation von Personen mit CI tatsächlich transformieren. Derartige Apps böten den Betroffenen nicht nur die Möglichkeit, die Einstellungen ihrer Geräte selbstständig an unterschiedliche Hörumgebungen anzupassen, sondern eröffneten auch den Zugang zu einer breiten Palette an Übungen und Feedback-Optionen. Darüber hinaus verbessern sie die Kommunikation zwischen den Patienten und ihren Audiologen, indem sie detaillierte Daten über die Nutzung des Implantats in einem gesicherten Datenraum nach dem Store-and-Forward-Prinzip bereitstellen. Dies fördert eine effektive, wenn auch indirekte Kommunikation. Dieser Ansatz, ergänzt durch die Eigeninitiative der Patienten bei Messungen und Anpassungen, entlastet die CI-Versorgungszentren und erlaubt den Nutzern gleichzeitig, ein tieferes Verständnis für ihr CI-System zu entwickeln.

Bekanntermaßen steigt mit zunehmendem Alter die Prävalenz von Hörverlusten signifikant an, wodurch altersbedingter Hörverlust zur Hauptindikation für CI wird. Dies stellte für die Verwendung von mHealth-Anwendungen lange Zeit eine Herausforderung dar, da traditionell die Verbreitung von Smartphones in älteren Bevölkerungsgruppen geringer war. Jedoch haben gerade die letzten Jahre, möglicherweise verstärkt durch die Pandemie, eine positive Entwicklung gezeigt: Immer mehr ältere Menschen nutzen Smartphones aktiv in ihrem Alltag. Laut einer Erhebung der Fa. Statista GmbH, Hamburg, Deutschland, nutzten im Jahr 2022 81,1 % der Bevölkerung in Deutschland regelmäßig ein Smartphone. Bei den über 70-Jährigen betrug der Anteil immer noch 68 %, sodass ein bedeutender Teil der Bevölkerung grundsätzlich für eine App-basierte Nachsorge infrage kommt [7].

Jeder Nutzer eines Smartphones kann effektiv die Kontrolle über Softwarefunktionen übernehmen

Die zunehmende Verbreitung von Smartphones in allen Altersgruppen ist auch ein Ergebnis der enormen Fortschritte in der Benutzerfreundlichkeit. Moderne Smartphones, die in ihrer Rechenleistung mit den Supercomputern der Jahrhundertwende vergleichbar sind, bieten intuitive Nutzeroberflächen, die eine leichte Bedienung ohne spezielle Vorkenntnisse ermöglichen. Dieser Fortschritt erleichtert es insbesondere älteren Menschen, sich mit der Technologie vertraut zu machen und sie für ihre Gesundheitsüberwachung zu nutzen. Damit eröffnen sich neue Möglichkeiten für eine individuell angepasste CI-Nachsorge, da jeder Nutzer eines Smartphones effektiv die Kontrolle über hochentwickelte Softwarefunktionen übernehmen kann.

Wesentliche Voraussetzung ist natürlich die Implementierung einer entsprechenden Funktion in die Hardware der CI-Prozessoren, die eine drahtlose Verbindung mit externen Geräten zulässt. Von Vorteil ist, dass die neueste Generation von CI-Prozessoren und Hörgeräten bereits Audio- und sogar Systemdaten mit bluetoothfähigen Geräten austauscht. Dies ermöglicht z. B. die Übertragung von Daten wie Elektrodenimpedanzen, Hautdicke (ermittelt über die Kopplungsgüte) und Hörgewohnheiten vom CI-Prozessor an ein Smartphone und deren weitere Übermittlung an ein zentrales gesichertes Datenbanksystem. Darüber hinaus könnten Patienten über eine Smartphone-App ihre Hörwahrnehmung verändern (Tiefen/Mitten/Höhen) und das situationsbedingt erstellte Hörprogramm für eine spätere Wiederverwendung abspeichern. Ebenso können Sprachtests mit dem Smartphone via Audiostreaming durchgeführt werden, was durch den rein digitalen Übertragungsweg bis zum Sprachprozessor eine kalibrierte Durchführung klinisch angewandter Sprachtests erlaubt.

Die Hörsystemhersteller arbeiten bereits an Lösungen, die diese wesentlichen, ärztlich-audiologisch notwendigen Funktionen enthalten. Vorreiter ist hier Cochlear Ltd, Sydney, Australien, mit der Funktion „Remote Check“. Der Patient kann damit auf dem Smartphone seine eigene Hörfähigkeit zumindest grob testen, indem er eine Aufblähkurve erstellt, einen Zahlentest im Störgeräusch durchführt und einen Fragebogen zum subjektiven Hörvermögen ausfüllt. Zusätzlich werden objektive Messparameter wie Elektrodenimpedanzen und das Logbuch des Klassifikators an eine zentrale Datenbank übertragen und für den Audiologen aufbereitet. Aber auch die Hersteller MED-EL (Innsbruck, Österreich) und Advanced Bionics AG (Stäfa, Schweiz) arbeiten an Lösungen, die zumindest schon im Rahmen von Studien eingesetzt werden und vom Funktionsumfang teilweise noch über die derzeitige Version von Remote Check hinausgehen.

Die Integration weiterer Parameter wie T‑ und C‑Werte („threshold“, Schwellenwert; „comfort“, gerade noch angenehme Lautstärke), Hautdicke und elektrisch ausgelöste Summenaktionspotenziale („electrically evoked compound action potentials“, ECAP) könnte zukünftig einen umfangreichen Datensatz (Big Data) für tiefere Einblicke in die individualisierte CI-Anpassung und Nutzerpräferenzen schaffen. Zudem könnte die Telemedizin die Patientensicherheit erhöhen, indem beispielsweise ein abnormer Anstieg der Impedanzwerte als potenzielle Entzündung in der Cochlea von KI-Algorithmen identifiziert und gemeldet wird.

Dieses erhöhte Sicherheitsniveau entspricht auch den Erwartungen der Kostenträger an die Telemedizin

Ähnliches gilt für die automatische Hautdickenmessung, die bei kontinuierlicher Abnahme auf eine zu hohe Magnetstärke hinweist. Dieses erhöhte Sicherheitsniveau durch Telemonitoring entspricht auch den Erwartungen der Kostenträger an die Telemedizin. In den nächsten Abschnitten werden die derzeitig in der Entwicklung befindlichen Lösungen der Hersteller Cochlear, Advanced Bionics und MED-EL dargestellt.

### „Remote Check“ der Fa. Cochlear

**Abb. 1:**
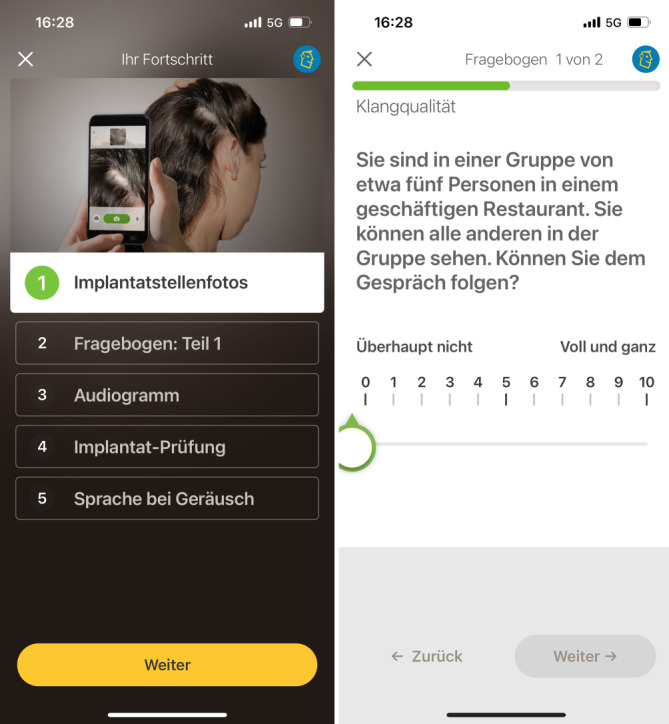
Screenshots aus der App Remote Check der Fa. Cochlear. *Links *die 5 Programmpunkte, die bei der Testsitzung vom Patienten durchlaufen werden müssen. *Rechts *Ausschnitt aus dem Fragebogen, der vom Patienten bei jedem Durchlauf ausgefüllt wird. (© Cochlear Ltd, Sydney, Australien)

Der 2019 eingeführte Remote Check von Cochlear repräsentierte einen bedeutenden Fortschritt in der autonomen Überwachung und Selbstverwaltung von CI-Trägern (Abb. 1). Diese App ermöglicht es CI-Nutzern erstmals, unabhängig von zu Hause aus die Funktion des Implantats sowie das Hörvermögen zu testen und die Ergebnisse für eine asynchrone Bewertung durch den Experten bereitzustellen. Das System ist grundsätzlich der Telemedizinkategorie Store-and-Forward zuzuordnen.

Um Remote Check nutzen zu können, sind einige Voraussetzungen zu erfüllen: Ein aktueller Soundprozessor (ab Nucleus® 7 oder Kanso® 2 Generation) ist erforderlich, ebenso ein kompatibles Smartphone. Eine Liste kompatibler Geräte findet sich auf der Cochlear-Webseite [8]. Die Nucleus® Smart-App, die Remote Check beinhaltet, muss auf dem Smartphone installiert sein und ist sowohl für Android als auch für iOS verfügbar. Der Patient benötigt zudem ein digitales Cochlear-Konto, welches auf myCochlear.com oder direkt in der Nucleus® Smart-App angelegt werden kann. Nach Kopplung des Soundprozessors mit der App und Aktivierung der Datensynchronisierung sind alle technischen Voraussetzungen erfüllt.

**Abb. 2:**
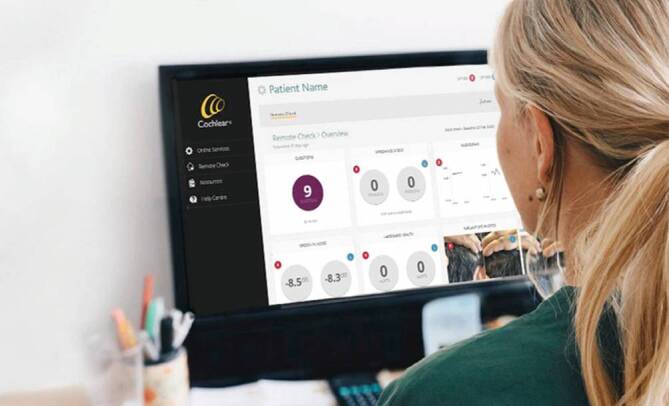
Professional Portal mit Übersicht der erhobenen Testdaten für den Audiologen. (© Cochlear Ltd, Sydney, Australien)

Die Aktivierung von Remote Check erfolgt durch einen Audiologen, wobei dies auch aus der Ferne möglich ist. Nach Erhalt einer Remote-Check-Benachrichtigung in der Smart-App hat der Patient 2 Wochen Zeit, die vorgegebenen Aufgaben und Tests zu absolvieren. Diese Tests können individuell an den Patienten angepasst und in der Nucleus® Smart-App durchgeführt werden. Nach Abschluss der Tests werden die Ergebnisse im myCochlear Professional Portal unter „Remote Check“ zur Überprüfung und Bewertung durch den Audiologen bereitgestellt (Abb. 2). Basierend auf den Ergebnissen können dann weitere Tests angeordnet oder eine Einladung des Patienten in die Klinik veranlasst werden, falls erforderlich.

Die Remote-Check-Testbatterie der Nucleus® Smart App umfasst die folgenden Tests, um die Funktionalität des CI, die Hörleistung und die subjektive Zufriedenheit zu überprüfen.

#### Aided Threshold Test

Der Aided Threshold Test (ATT, Aufblähkurve) sollte in einer ruhigen Umgebung durchgeführt werden, um eine genaue Messung der Hörschwellen zu ermöglichen, wichtig für Nutzer mit Restgehör (Nutzer der elektrisch-akustischen Stimulation [EAS] oder bimodale Nutzer). Er beinhaltet das Erkennen von reinen Tönen, die direkt vom Smartphone gestreamt werden, und gibt ein vollständiges Audiogramm über die Sprachfrequenzen.

Der Algorithmus startet bei einem Präsentationspegel von 40 dB HL und verwendet ein adaptives Stufenverfahren mit variabler Schrittgröße, um den Schwellenwert für jede Frequenz zu ermitteln. Zu Beginn des Tests wird eine Schrittgröße von 8 dB verwendet, die Schrittgröße wird sukzessive auf ein Minimum von 1 dB verringert, wenn sich der Stimulus dem Schwellenwert nähert [9].

#### Digit Triplet Test

Der Digit Triplet Test (DTT) ist ein adaptiver Test, der das Verstehen von Zahlen im Geräusch misst. Das Signal-Rausch-Verhältnis (SNR) wird basierend auf den Antworten des Nutzers kontinuierlich anpasst, jedoch liegt der kombinierte Pegel immer bei 65 dB SPL. Der Testmodus beginnt bei −6 dB SNR und wird basierend auf den Antworten des Teilnehmers um ± 2 dB angepasst. Es werden 2 Sets mit jeweils 8 Zahlentriplets von 1 bis 9 präsentiert, z. B. 1‑3‑8. Die Zahl 7 ist wegen ihrer Zweisilbigkeit ausgeschlossen. Der durchschnittliche SNR der insgesamt 16 Triplets wird als SRT (Sprachverständlichkeitsschwellenwert) aufgezeichnet. Ergibt sich ein Unterschied zwischen den 2 präsentierten Sets von mehr als 3 dB, wird das Testergebnis als unzuverlässig im Professional Portal angezeigt.

#### Fragen zum allgemeinen Gesundheitszustand und zur Hör‑/Klangqualität

Es gibt eine Erwachsenen- und eine Elternversion des Fragebogens mit jeweils 30 bzw. 38 Fragen, die vom Patienten oder dessen Betreuer/Eltern, je nach Alter, ausgefüllt werden. Die Fragebögen enthalten Fragen zu medizinischen Aspekten (im Wesentlichen Fragen zu eventuellen Schmerzen im Bereich des Implantats), Anpassungsparametern, Gerätekomponenten und Klangqualität. Der validierte SSQ-Fragebogen (Speech, Spatial and Qualities of Hearing Scale; Sprachverstehen, räumliches Hören, Hörqualität) [10] zur Erfassung der subjektiven Hörleistung ist Bestandteil des Fragenkatalogs. Der Erwachsenen-Fragebogen enthält die 12 Fragen aus dem kurzen SSQ12-Fragebogen [11], der Eltern-Fragebogen enthält die 23 Fragen aus dem SSQ für Eltern [12]. Beim Beantworten der SSQ-Fragen, die auf einer Skala von 1 bis 10 bewertet werden, erhalten die Befragten ihre vorangegangene Bewertung des letzten Termins, um das Antworten zu erleichtern. Auch gibt es die Option, zusätzliche Informationen bereitzustellen, falls der Patient Probleme melden möchte.

Die Antworten auf den Fragebogen werden vom Kliniker überprüft, um Hörstatus und Probleme zu ermitteln

Die Antworten auf den Fragebogen werden vom Kliniker überprüft, um den aktuellen Hörstatus und eventuelle Probleme zu ermitteln, die weitere Maßnahmen einschließlich eines Klinikbesuchs erfordern. Typischerweise wird für CI-Patienten ein Unterschied in den SSQ-Bewertungen zwischen den Testintervallen von > 1 als klinisch relevant angesehen [13].

#### Data Logging

Die Sammlung von Nutzungsdaten bietet Einblicke in das tägliche Nutzungsverhalten und zeigt damit mögliche Probleme in Bezug auf die Programmierung und das Hörvermögen des Patienten an, etwas, das deutlich über die Möglichkeiten der herkömmlichen Nachsorge hinausgeht. Der Soundprozessor speichert Daten über die Nutzungsdauer, das Abnehmen der Spule (Verbindungsverlust), die Verwendung der unterschiedlichen Programme und Zubehör, Lautstärkeeinstellungen, Empfindlichkeits-, und Mikrofoneinstellung und die Dauer des Aufenthalts in verschiedenen akustischen Umgebungen. Die Nucleus® Smart App erfasst stündlich einen Schnappschuss der Nutzungsdaten, wenn sich das Smartphone in Reichweite des Prozessors befindet. So können Hörprobleme über Verhaltensänderungen der Patienten ermittelt werden, etwa anhand einer zunehmenden Vermeidung von geräuschvollen Umgebungen. Die Nutzungsdaten werden im Cochlear Professional Portal dargestellt, einerseits als Mittelwerte wie auch in der klinischen Fittingsoftware Custom Sound, andererseits auch als detaillierte Tagesansicht für Nutzungsdauer, Aufenthalt in unterschiedlichen akustischen Szenarien, die Verwendung unterschiedlicher Mikrofoneinstellungen und des Zubehörs. Ebenfalls werden eventuelle Probleme mit dem Mikrofon oder des Prozessors angezeigt, basierend auf den gespeicherten Ergebnissen der Selbsttests des Soundprozessors.

#### Impedanzmessungen

Eine automatisierte Impedanztelemetrie wird durchgeführt, um die Integrität der Elektrode-Nerv-Schnittstelle zu überprüfen. Neben den Impedanzwerten für alle Elektroden werden offene oder kurzgeschlossene Elektroden angezeigt. Starke Abweichungen der Impedanzwerte zum vorangegangenen Termin können ein Hinweis auf intracochleäre Entzündungen sein [14] und auf die Notwendigkeit einer Anpassung der Stimulationsparameter [15] bzw. eines Klinikbesuchs hinweisen. Diese Werte werden, wie die Ergebnisse der anderen Tests, im myCochlear Professional Portal für den Kliniker dargestellt.

#### Fotos vom Implantatbereich

Bei diesem Schritt erstellen Patienten selbst oder, im Idealfall, eine andere Person, Fotos von der Haut im Bereich des Implantats und von der Operationsnarbe hinter dem Ohr. Die App bietet hierfür eingebaute Anleitungen und Beispielbilder, um eine korrekte Durchführung zu gewährleisten. Zudem ermöglicht sie das Wiederholen der Aufnahmen bei Bedarf. Diese Fotos werden anschließend vom Kliniker, zusammen mit den Antworten aus dem Fragebogen zur Hautgesundheit, ausgewertet. Dies dient der Erkennung von möglichen Hautentzündungen, Reizungen oder anderen Komplikationen im Bereich des Implantats.

#### Anpassung von Hörprogrammen

Wenn Patienten ihre Hörprogramme (MAP) anpassen möchten, bietet die App „mySmartSound“, die wie „Remote Check“ Teil der Nucleus® Smart-App ist, diese Möglichkeit in einem gewissen Rahmen. „mySmartSound“ ermöglicht es Patienten, die C‑Level durch Funktionen wie Master Volume, Bässe und Höhen eigenständig zu justieren. Diese Funktion, die vom Audiologen freigeschaltet werden muss, eignet sich besonders für erfahrene Patienten mit stabilen Einstellungen. Das Konzept ähnelt der von Smoorenburg beschriebenen „Shift-und-Tilt-Methode“ [16]: Mit den Reglern für Höhen und Bässe wird eine Kippung (Tilt) des ursprünglichen C‑Level-Profils erreicht, während mit dem Master Volume eine Verschiebung (Shift) des C‑Level-Profils nach oben oder unten erfolgt.

Der Patient hört die Auswirkungen seiner Einstellung unmittelbar in der entsprechenden Hörsituation

Der Patient kann diese Anpassungen an allen im Prozessor gespeicherten MAP vornehmen und hört die Auswirkungen seiner Einstellung unmittelbar in der entsprechenden Hörsituation. Sind auf den 4 Programmspeichern des Prozessors unterschiedliche MAP gespeichert, so sind Änderungen auf jedem einzelnen Programmplatz möglich. Diese Anpassungen werden beim nächsten Besuch im CI-Zentrum beim Anschluss des Prozessors an Custom Sound automatisch in das Programm übertragen und können dort vom Audiologen weiterverarbeitet werden.

### „Research-App“ der Fa. Advanced Bionics

Advanced Bionics entwickelt ebenfalls eine innovative App, die Nutzern von CI eine selbstständige Überprüfung und in gewissem Rahmen auch die Anpassung ihrer Hörprogramme ermöglicht. Aktuell ist diese App jedoch ausschließlich als Forschungsinstrument („Research-App“) verfügbar und kann daher nur im Rahmen wissenschaftlicher Studien genutzt werden. Das aktuelle System umfasst 2 separate Apps: eine für die Überprüfung und Einstellung des Implantats und eine weitere für die Selbsttestung von Hörschwellen und Sprachverständnis.

Ein signifikanter Unterschied zum Konzept des Wettbewerbers Cochlear ist, dass die Nutzer der Advanced-Bionics-Apps diese unabhängig und so oft wie gewünscht verwenden können, ohne auf eine Einladung des Audiologen angewiesen zu sein. Besonders hervorzuheben ist die Möglichkeit, die App auch bei bimodaler Versorgung einsetzen zu können. Dies ermöglicht es auch Patienten, die sowohl ein CI als auch ein Hörgerät nutzen, ebenfalls eigene Hörprogramme über die App zu erstellen und zu speichern.

In den nachfolgenden Abschnitten werden die spezifischen Funktionen und Möglichkeiten dieser App detailliert erörtert.

#### Test des Gerätezustands

Die App ermöglicht es, den Sprachprozessor einem Selbsttest zu unterziehen. Dabei werden die integrierten Mikrofone sowie ein eventuell vorhandenes T‑Mic-Mikrofon auf ihre Übertragungsqualität geprüft. Zusätzlich wird die Verbindung zwischen dem Sprachprozessor und dem Implantat überwacht und deren Kopplungsgüte angezeigt.

#### Überprüfung der Hautdicke

Eine besonders nützliche Funktion der App ist die Überwachung der Hautdicke (Magnetcheck-Funktion) zwischen der externen Spule und dem Implantat. Häufig ist es notwendig, anfänglich einen stärkeren Magneten in der externen Spule zu verwenden, um einen sicheren Halt auf der Haut zu gewährleisten. Allerdings kann der anhaltende Druck zwischen externer Spule und Implantat über die Zeit zu einer Reduzierung der Hautdicke führen, was eine Anpassung der Magnetstärke erfordert. Wird dies nicht beachtet, kann es zu einer weiteren Reduktion der Hautdicke und im schlimmsten Fall zu einer Nekrotisierung des Gewebes kommen. Die App misst kontinuierlich die Qualität der Kopplung zwischen der internen und externen Spule und kann somit frühzeitig geringfügige Veränderungen des Spulenabstands erkennen. Aktuell werden die erhobenen Daten auf einem gesicherten Server gespeichert.

Ziel ist es, aus den erhobenen Daten kritische Schwellwerte zu bestimmen

Ziel ist es, aus den erhobenen Daten kritische Schwellwerte zu bestimmen, sodass künftig bei einer fortschreitenden Hautdickenveränderung Warnmeldungen an den Patienten oder Audiologen gesandt werden können.

#### Anpassung des Hörprogramms

**Abb. 3:**
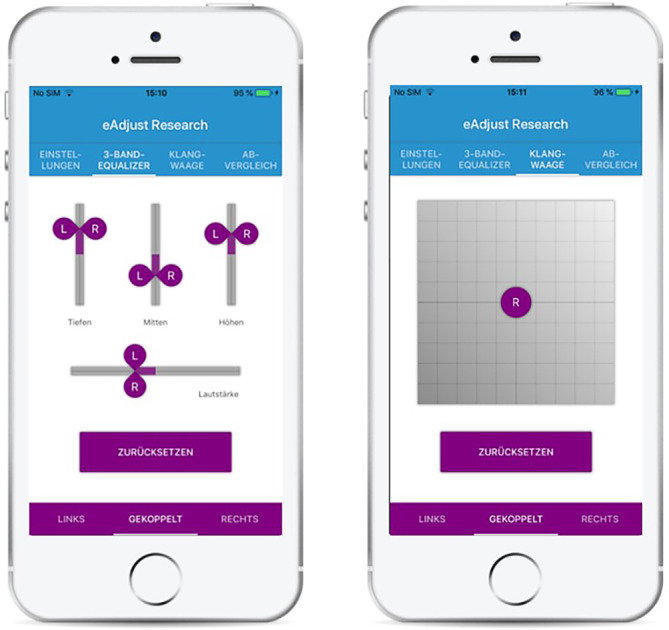
Darstellung der beiden Oberflächen zur Einstellung der Klangwahrnehmung. *Links* klassischer Ansatz mittels 3‑Band-Equalizer (Tiefen, Mitten, Höhen), *rechts *2‑D-Touchoberfläche mit der Möglichkeit einer Klangveränderung durch Verschieben des *Kreises* innerhalb der Fläche. (Aus [17] © S. Kliesch et al., CC BY 4.0; https://creativecommons.org/licenses/by/4.0/)

Die App bietet 2 verschiedene Benutzeroberflächen, mit denen Patienten ihre Klangwahrnehmung an die aktuelle Hörumgebung anpassen können (Abb. 3).

**Abb. 4:**
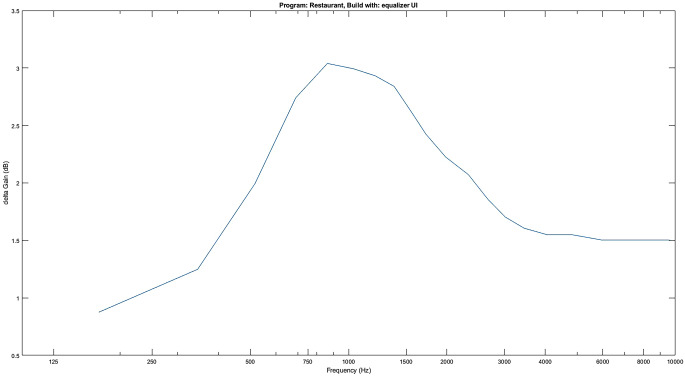
Beispiel einer Anpassung der Gains über die Equalizer-Oberfläche eines Patienten bei einem Restaurantbesuch. Anhebung der Frequenzen im Bereich 500–3000 Hz um bis zu 3 dB und leichte Anhebung der Höhen um 1,5 dB

Die vorgenommenen Einstellungen wirken sich direkt auf die Gains der 16 Bandpassfilter aus (Abb. 4). Nach der Feinabstimmung kann das individuelle Programm unter einem spezifischen Namen gespeichert und bei Bedarf wieder aufgerufen werden.

Im Rahmen einer derzeit laufenden Studie wurden bisher 15 Patienten mit den Apps ausgestattet und konnten damit eigene Einstellungen vornehmen und Hörtests durchführen. Vorangegangene Studien zeigen, dass solche Anpassungen unter kontrollierten Laborbedingungen überwiegend zu einer subjektiven Verbesserung des Höreindrucks führen und auch eine derartige Einstellmöglichkeit an sich positiv bewertet wird [17].

Die ersten Daten offenbaren, dass die App von einer signifikanten Anzahl der Teilnehmer während des gesamten Studienzeitraums von 6 Monaten aktiv genutzt wurde, wobei regelmäßig individuelle Anpassungen im Alltagskontext vorgenommen wurden. Die von den Teilnehmern getroffenen Einstellungen werden, ähnlich wie unter kontrollierten Laborbedingungen [17], positiv bewertet. Auffällig ist, dass die meisten Anpassungen in Situationen mit sprachbezogenem Kontext erfolgten. Mit Stand November 2023 hatten 14 Studienteilnehmer die 6‑monatige Studie komplett durchlaufen. Es zeigte sich, dass während der Studiendauer im Mittel von jedem Teilnehmer 12 Hörprogramme für unterschiedliche Situationen erstellt und diese Programme mehrfach vom Patienten wieder aufgerufen wurden.

#### Klinisch relevante Sprachtests

Die App integriert audiologische Tests wie die Bestimmung der Hörschwelle, einen Phonemtest und den Oldenburger Satztest (OlSa). Der OlSa ist besonders vorteilhaft, da er ein klinisch validierter, mehrsprachiger Satztest ist, der weltweit eingesetzt werden kann und den Anforderungen des Weißbuchs Cochlea-Implantat(CI)-Versorgung (CI-Weißbuch) sowie des Deutschen CI-Registers entspricht.

Die Tests können direkt über Bluetooth vom Smartphone oder Tablet an den Prozessor übertragen werden

**Abb. 5:**
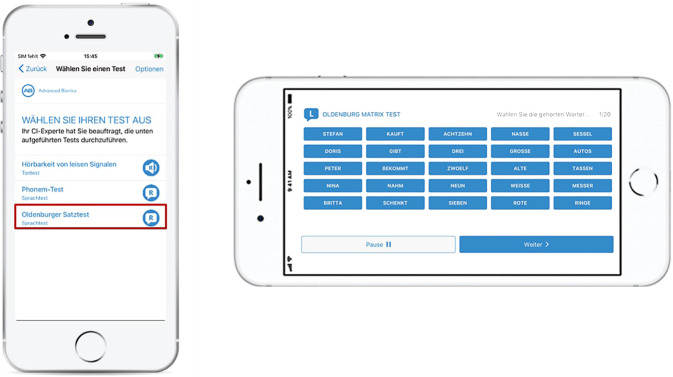
Oberfläche der Testauswahl (*links*) und der Antwortmatrix des Oldenburger Satztests (*rechts*) in der Advanced-Bionics-Forschungs-App („Research-App“). (© Advanced Bionics AG, Stäfa, Schweiz)

Die Tests können direkt über Bluetooth vom Smartphone oder Tablet an den Prozessor übertragen werden, wodurch sie in kalibrierter Form und selbst in akustisch ungünstigen Umgebungen durchgeführt werden können. Bei Patienten mit Restgehör sollte jedoch eine ruhige Umgebung zur Durchführung des Tests aufgesucht werden. In der derzeitigen Implementierung werden die Antworten des Patienten über eine Touchoberfläche aus einer vorgegebenen Wortliste ausgewählt (Abb. 5).

Zukünftige Versionen könnten eine Funktion enthalten, bei der Patienten die gehörten Wörter einfach nachsprechen – vergleichbar mit der Situation beim klinischen Test. Auf dem Smartphone kommt dann ein automatischer Spracherkenner zum Einsatz, der die Antworten des Nutzers bewertet. Die Machbarkeit dieser Methode wurde bereits im Rahmen des Hearing4all-Exzellenzclusters durch eine Implementierung des OlSa auf Amazon Alexa demonstriert [18].

### Remote-Care-Konzept der Fa. MED-EL

Im Bereich der Remote-Care-Lösungen für CI-Patienten ist MED-EL ein weiterer wichtiger Akteur, der innovative Konzepte entwickelt. Ähnlich wie die Hersteller Advanced Bionics und Cochlear arbeitet auch MED-EL an einem Nachsorgekonzept, das es Patienten ermöglicht, über ein Smartphone selbstständig Daten zu erheben und diese mit ihrem CI-Zentrum zu teilen. Dieses Konzept zielt darauf ab, die Anzahl der Zentrumsbesuche zu reduzieren und gleichzeitig eine kontinuierliche und lückenlose Datenerhebung zu gewährleisten. Geplant sind u. a. die Messung objektiver Parameter wie Elektrodenimpedanzen und Performancedaten zur Hörentwicklung. Besonderes Augenmerk legt MED-EL darauf, dass das für 2024 geplante Softwarekonzept den Vorgaben des CI-Weißbuchs der Deutschen Gesellschaft für Hals-Nasen-Ohren-Heilkunde, Kopf- und Hals-Chirurgie e.V. (DGHNO-KHC) entspricht, um die erhobenen Daten auch für Registerzwecke nutzbar zu machen.

In der Vergangenheit hat MED-EL, ähnlich wie andere Hersteller, explorative Forschungs-Apps im Feld getestet, die speziell für die Messung und Übertragung von Impedanzdaten sowie für die Selbsttestung der Hörleistung von Patienten konzipiert wurden. Im Folgenden wird detailliert auf die „Remote-Check-App“ zur Selbstüberprüfung der Hörleistung und auf die Telemetrie-App zur Messung der Elektrodenimpedanzen eingegangen.

#### Telemetrie-App

Obwohl ursprünglich für Forschungszwecke entwickelt, ist geplant, das Konzept der Telemetrie-App in eine klinisch zugelassene Version zu integrieren. Dies begründet sich in den wertvollen Erkenntnissen, die durch die Nutzung der App gewonnen wurden. Kompatibel mit iOS-Smartphones, ermöglichte die App tägliche Impedanzmessungen der intracochleären Elektroden, um den Zustand des Innenohrs zu überwachen. Ein Anstieg der Elektrodenimpedanz kann auf eine Entzündungsreaktion im Innenohr hinweisen, die sowohl unmittelbar bzw. Tage nach der Elektrodeninsertion als auch durch verschiedene andere Ursachen noch Monate oder Jahre später auftreten kann.

Entzündungen können durch regelmäßige Impedanzmessungen frühzeitig erkannt werden

**Abb. 6:**
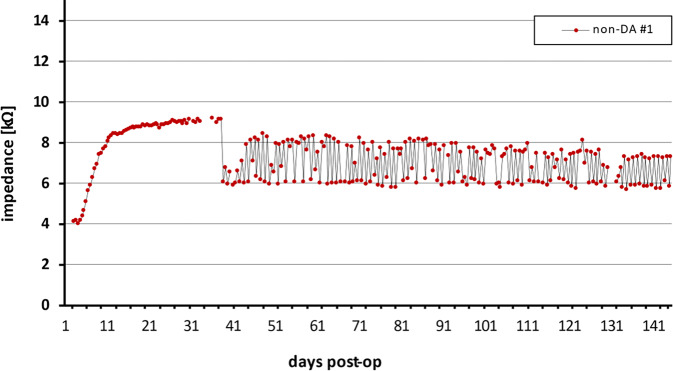
Vom Patienten durchgeführte Impedanzmessungen, zum Teil mehrfach täglich, beginnend einen Tag nach Op. für einen Zeitraum von über 3 Monaten. Gut sichtbar der Anstieg der Impedanzen in der postoperativen Heilungsphase ohne Stimulation und der deutliche Abfall der Impedanzwerte am Tag 41 bei Erstaktivierung des Cochlea-Implantat(CI)-Systems. In der Folge Impedanzschwankungen (*Zickzackmuster*) morgens/abends gut sichtbar, mit höheren Impedanzen am Morgen

Diese Entzündungen können durch regelmäßige Impedanzmessungen frühzeitig erkannt werden. Die Möglichkeit, tägliche In-vivo-Impedanzmessungen durchzuführen, bietet damit einen deutlichen Sicherheitsvorteil im Vergleich zur derzeitigen klinischen Praxis mit jährlichen Kontrollterminen. Während laufender Studien konnten Patienten die Impedanzen nach Belieben messen, wobei die Daten automatisch auf einen sicheren Server übertragen wurden. Die erhöhte Messfrequenz erlaubt es, das Verhalten der Impedanz langfristig genauer zu analysieren (Abb. 6).

#### „Remote Check“

Die Forschungs-App „Remote Check“ der Fa. MED-EL, Innsbruck, Österreich, diente ausschließlich dazu, ein auditorisches Profil und damit die Hörleistung des Patienten zu bestimmen. Eine umfassende technische Überprüfung des CI-Systems ist jedoch nicht in den Funktionsumfang der App integriert.

**Abb. 7:**
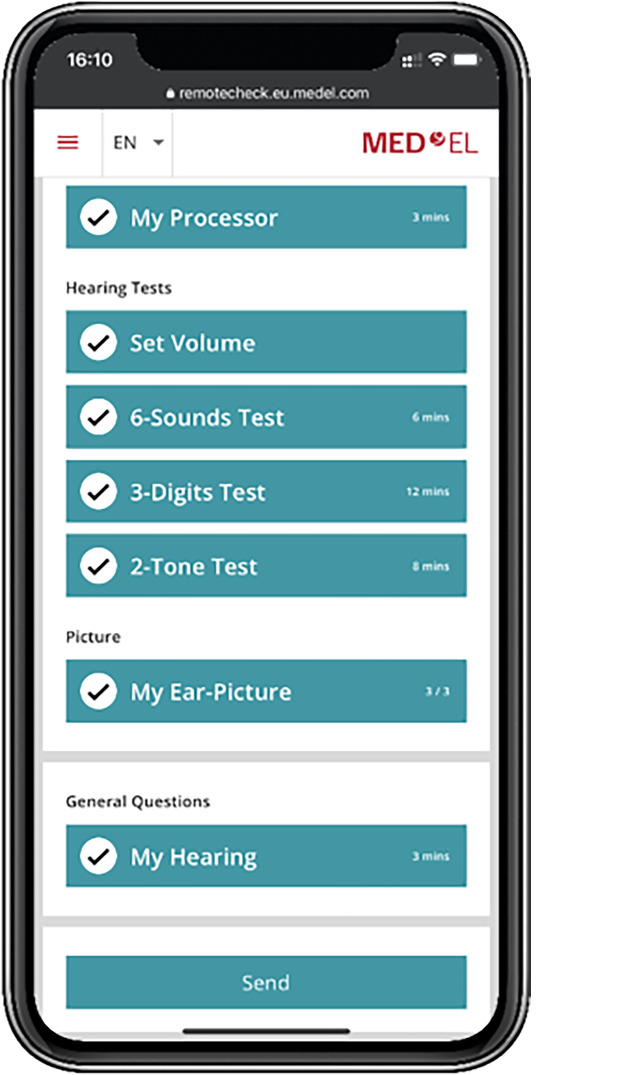
Screenshot der MED-EL-Remote-Check-App, die der Patient schrittweise durchläuft. (© MED-EL, Innsbruck, Österreich)

In Abb. 7 sind die vom Patienten auszuführenden Schritte dargestellt, um den Selbsttest vollständig durchzuführen. Als Grundvoraussetzung für den Test muss der CI-Prozessor mit dem Smartphone verbunden sein. Dies ermöglicht es, die Audiosignale an den Prozessor zu übermitteln. Je nach Modell des Prozessors wird entweder eine analoge Verbindung mittels Audiokabel oder eine drahtlose Verbindung via Bluetooth genutzt, wobei ggf. der AudioLink von MED-EL zum Einsatz kommt. Zudem ist es erforderlich, dass der Patient die Lautstärke des Smartphones auf das Maximum einstellt. Anleitungen hierzu werden auf dem Smartphone-Bildschirm angezeigt. Nach diesen Vorbereitungen beginnen die Hörtests.

#### 6-Laute-Test

**Abb. 8:**
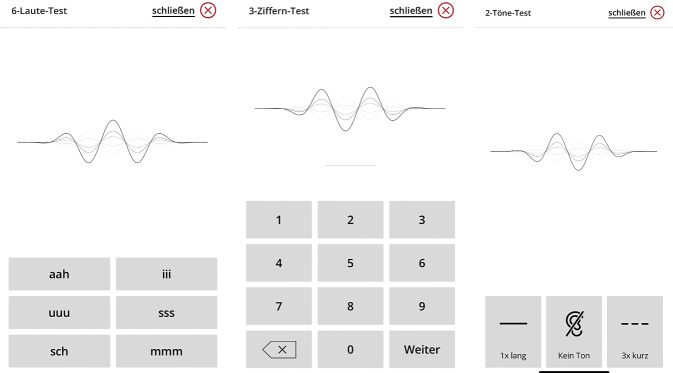
Darstellung des Smartphone-Bildschirms für die Aufgaben „6-Laute-Test“ (*links*), „3-Ziffern-Test“ (*Mitte*) und „2-Töne-Test“ (*rechts*)

Bei diesem Test werden 6 synthetisch generierte Ling-Laute (Ling Six Sound Test) vorgespielt. Diese sind „ahh“, „iii“, „uuu“, „sss“, „sch“, „mmm“ (Abb. 8). Jeder der Laute wird 6‑mal in randomisierter Folge dargeboten. Der Präsentationspegel wird über den gesamten Test konstant gehalten, es wird kein Hintergrundgeräusch präsentiert. Auf dem Bildschirm werden dem Patienten 6 Auswahlmöglichkeiten gezeigt, der Patient tippt auf die von ihm wahrgenommene Variante.

#### 3-Ziffern-Test

Bei diesem Test werden, sehr ähnlich zum DTT im Remote Check von Cochlear, 3 zufällig ausgewählte Ziffern vorgesprochen, die der Patient über die Tastatur wiederholen soll. Es handelt sich um einen adaptiven Test im Störgeräusch. Das Verhältnis zwischen Nutz- und Störsignal wird solange angepasst, bis der Patient ein 50%iges Zahlenverstehen erreicht hat. Bei der Implementierung des Tests orientierte sich MED-EL an einer entsprechenden Arbeit von Smits et al. [19].

#### 2-Töne-Test

Der Test zielt auf die Bestimmung der Hörschwelle bzw. Aufblähkurve ab. MED-EL verwendet dafür das Duoton-Verfahren [20]. Diese Methode setzt Paare von Reintonsignalen zur Ermittlung der Hörschwelle ein. Jedes Paar besteht aus einem tieferen Ton von 700 ms Dauer, gefolgt von einer kurzen Pause und dann 3 höheren Tönen gleicher Frequenz, von denen jeder 220 ms dauert. Zusätzlich wird dem Nutzer ein drittes, „stilles Signal“ präsentiert. Nach jedem zufällig ausgewählten Stimulus tippt der Patient auf eine von 3 Tasten, die dem wahrgenommenen Signal entspricht, wie in Abb. 8 dargestellt.

**Abb. 9:**
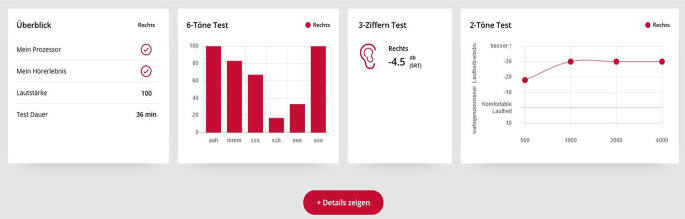
Anzeige der Testdaten eines Studienpatienten, der einen kompletten Durchlauf mit der App „Remote Check“ der Fa. MED-EL, Innsbruck, Österreich, durchlaufen hat. Darstellung für den Audiologen, kein Zugriff des Patienten selbst auf die Ergebnisse

Nach Abschluss der Sequenz berechnet die App den Hörschwellenwert, und anschließend wird ein weiteres Frequenzpaar getestet (Abb. 9). Insgesamt werden 6 Frequenzpaare getestet, die einen Bereich von 125 Hz bis 8 kHz abdecken.

#### Foto der Implantatstelle und des Ohrs

Nach Abschluss der audiologischen Tests wird vom Patienten oder einer Hilfsperson ein Foto der Haut über dem Implantat aufgenommen. Zusätzlich soll ein Bild des Gehörgangs erstellt werden.

Diese Aufnahmen dienen der Beurteilung der Hautgesundheit durch Fachpersonal des CI-Zentrums. Die Fotos werden sicher gespeichert und können in einem geschützten Online-Bereich eingesehen werden.

#### Fragebögen zum Hörverständnis und Tragekomfort des Prozessors

Die App beinhaltet auch einen Abschnitt, in dem der Patient Fragen zu seiner Hörfähigkeit und dem Tragekomfort des Prozessors beantworten muss. Bei den Fragen zur Hörfähigkeit geht es um das Verständnis in ruhigen sowie in lauten Umgebungen sowie um das Verstehen beim Telefonieren. Die Fragen zum Tragekomfort konzentrieren sich auf eventuelle Hautirritationen oder Schmerzen, die beim Tragen des Prozessors auftreten können.

## Ausblick

Die App-basierte Nachsorge von CI-Patienten als Alternative zu einem Besuch im CI-Zentrum befindet sich in greifbarer Nähe. Die Menge an Daten, die durch solche Apps generiert wird, ist beispiellos in der CI-Versorgung, und es ist zu erwarten, dass insbesondere aus den häufig und automatisiert gemessenen Impedanzdaten, die dann von Tausenden von Patienten weltweit verfügbar wären, viele neue Erkenntnisse zum Zustand des Innenohrs – gerade auch im Zusammenhang mit dem Erhalt von akustischem Restgehör –gezogen werden können.

Die Möglichkeit für Patienten, Hörprogramme in verschiedenen Situationen selbst anzupassen und zu speichern, ist weit mehr als nur eine Spielerei. Die Funktion wurde im Rahmen von mehrmonatigen Studien von Patienten deutlich häufiger genutzt als erwartet. Zusammen mit den Daten des Hörsituationsklassifikators können zukünftig aus Tausenden von individuellen Patientenprogrammen wichtige Erkenntnisse gewonnen werden, um Patienten in unterschiedlichen Hörsituationen mit dem besten Programm automatisiert zu versorgen. Bemerkenswert ist, dass auch hochbetagte Patienten im Rahmen der durchgeführten Studien gut mit den getesteten Apps zurechtkamen; vorausgesetzt, sie hatten bereits Erfahrung mit der Benutzung von Smartphones. Die digitalen Lösungen bieten damit insgesamt einen erheblichen Mehrwert für die Nachsorge, indem sie eine kontinuierliche und personalisierte Versorgung bei gestiegener Patientensicherheit ermöglichen.

Die Integration von KI in Verbindung mit stark wachsenden Datenmengen, erhoben unter den Vorgaben der aktuellen Datenschutzgesetze, wird zweifellos zu individuelleren Empfehlungen und personalisierten Anpassungen führen, ohne das Fachpersonal zusätzlich zu belasten. Ferner eröffnet die Möglichkeit der Fernüberwachung und -diagnose gerade kranken Patienten oder solchen in abgelegenen oder unterversorgten Gebieten die Chance, umfassend von einer engmaschigen CI-Nachsorge zu profitieren. Erneut hervorzuheben ist an dieser Stelle die Notwendigkeit, für die CI-Nachsorge relevante Sprachtests und Datenerfassungen in die Apps zu integrieren. So ist die Implementierung eines anerkannten, klinisch angewandten Sprachtests im Störgeräusch, der auch für die Erfüllung der Weißbuchvorgaben zur CI-Nachsorge und für das CI-Register notwendig ist, aus audiologischer Sicht unabdingbar. Dies könnte zudem die Möglichkeit eröffnen, über die Apps Studienmessungen durchzuführen, wie beispielsweise den Vergleich neuer Sprachverarbeitungsstrategien bei der Bereitstellung neuer Firmware für den Prozessor – mit dem OlSa selbst über Sprachgrenzen hinweg. Auch wenn gerade Letztgenanntes derzeit vielleicht noch utopisch klingen mag, wäre es technisch bereits mit den heutigen CI-Systemen umsetzbar.

Abschließend lässt sich feststellen, dass die fortschreitende Entwicklung und Anwendung dieser Apps nicht nur die Lebensqualität von CI-Patienten deutlich verbessert, sondern auch neue Wege in der personalisierten Medizin eröffnet. Diese Entwicklung verspricht, die Ressourceneffizienz in der CI-Versorgung zu steigern und gleichzeitig das Patientenwohl durch verstärkte Autonomie, höhere Sicherheit und individuell zugeschnittene Hörerlebnisse zu fördern.

## Fazit für die Praxis

- Die vorgestellten Ansätze der Hersteller zur App-basierten Nachsorge von Cochlea-Implantat(CI)-Patienten bieten eine Reihe an Funktionen, die sowohl für Patienten als auch für medizinisches Fachpersonal von wesentlicher Bedeutung sind und über bisherige Nachsorgekonzepte hinausgehen.
- Sie ermöglichen eine tägliche Überwachung des Implantatzustands, individuelle Anpassungen der Hörprogramme, audiologische Selbsttests und eine asynchrone, effektive Kommunikation mit dem behandelnden CI-Zentrum.
- Besonders hervorzuheben ist die grundsätzliche Möglichkeit, wichtige Daten wie Elektrodenimpedanzen in nahezu beliebiger Häufigkeit zu erfassen, um frühzeitig potenzielle Probleme zu erkennen und darauf zu reagieren.
- Hier ist auch die automatische, derzeit noch experimentelle Hautdickenmessung hervorzuheben, die einer Schädigung der Haut durch zu starke Magnetkraft vorbeugt, indem das CI-Zentrum rechtzeitig benachrichtigt wird.
- Diese Monitoringfunktionen sind es, die Kostenträger von der App-basierten Nachsorge erwarten, um zukünftig Komplikationen weiter zu reduzieren und damit die CI-Versorgung effizienter zu gestalten.
- Rückmeldungen von Studienteilnehmern bestätigen zudem einen in anderen medizinischen Bereichen beobachteten Vorteil des Patient Empowerments: eine gesteigerte Patientenzufriedenheit.
- Allein die Möglichkeit, einfach Einfluss auf das eigene Hörvermögen zu nehmen, steigert die Zufriedenheit der Patienten – selbst in Fällen, in denen keine objektive Hörverbesserung durch die selbst vorgenommenen Einstellungen nachweisbar ist.
